# Methicillin-Susceptible Staphylococcus aureus Biofilm Formation on Vascular Grafts: an *In Vitro* Study

**DOI:** 10.1128/spectrum.03931-22

**Published:** 2023-02-07

**Authors:** Cristina Tello-Díaz, Marta Palau, Estela Muñoz, Xavier Gomis, Joan Gavaldà, Nuria Fernández-Hidalgo, Sergi Bellmunt-Montoya

**Affiliations:** a Department of Vascular and Endovascular Surgery, Hospital de la Santa Creu i Sant Pau, Institute of Biomedical Research (II-B Sant Pau), CIBER CV, Barcelona, Spain; b Universitat Autònoma de Barcelona (UAB), Departament de Cirurgia i Ciències Morfològiques, Barcelona, Spain; c Antimicrobial Resistance Laboratory, Vall d'Hebron Research Institute (VHIR), Infectious Diseases Department, Hospital Universitari Vall d'Hebron, Barcelona, Spain; d CIBER de Enfermedades Infecciosas (CIBERINFEC), Instituto de Salud Carlos III (ISCIII), Madrid, Spain; e Infectious Diseases Department, Hospital Universitari Vall d’Hebron, Universitat Autònoma de Barcelona, Barcelona, Spain; f Red Española de Investigación en Patología Infecciosa (REIPI RD16/0016/0003), Instituto de Salud Carlos III, Madrid, Spain; g Department of Angiology, Vascular and Endovascular Surgery, Hospital Universitari Vall d’Hebron, Barcelona, Spain; Innovations Therapeutiques et Resistances

**Keywords:** vascular graft, infection, biofilm, *Staphylococcus aureus*, *in vitro*, prosthesis infections

## Abstract

The aim of this study was to quantify *in vitro* biofilm formation by methicillin-susceptible Staphylococcus aureus (MSSA) on the surfaces of different types of commonly used vascular grafts. We performed an *in vitro* study with two clinical strains of MSSA (MSSA2 and MSSA6) and nine vascular grafts: Dacron (Hemagard), Dacron-heparin (Intergard heparin), Dacron-silver (Intergard Silver), Dacron-silver-triclosan (Intergard Synergy), Dacron-gelatin (Gelsoft Plus), Dacron plus polytetrafluoroethylene (Fusion), polytetrafluoroethylene (Propaten; Gore), Omniflow II, and bovine pericardium (XenoSure). Biofilm formation was induced in two phases: an initial 90-minute adherence phase and a 24-hour growth phase. Quantitative cultures were performed, and the results were expressed as log_10_ CFU per milliliter. The Dacron-silver-triclosan graft and Omniflow II were associated with the least biofilm formation by both MSSA2 and MSSA6. MSSA2 did not form a biofilm on the Dacron-silver-triclosan graft (0 CFU/mL), and the mean count on the Omniflow II graft was 3.89 CFU/mL (standard deviation [SD] 2.10). The mean count for the other grafts was 7.01 CFU/mL (SD 0.82). MSSA6 formed a biofilm on both grafts, with 2.42 CFU/mL (SD 2.44) on the Dacron-silver-triclosan graft and 3.62 CFU/mL (SD 2.21) on the Omniflow II. The mean biofilm growth on the remaining grafts was 7.33 CFU/mL (SD 0.28). The differences in biofilm formation on the Dacron-silver-triclosan and Omniflow II grafts compared to the other tested grafts were statistically significant. Our findings suggest that of the vascular grafts we studied, the Dacron-silver-triclosan and Omniflow II grafts might prevent biofilm formation by MSSA. Although further studies are needed, these grafts seem to be good candidates for clinical use in vascular surgeries at high risk of infections due to this microorganism.

**IMPORTANCE** The Dacron silver-triclosan and Omniflow II vascular grafts showed the greatest resistance to *in vitro* methicillin-susceptible Staphylococcus aureus biofilm formation compared to other vascular grafts. These findings could allow us to choose the most resistant to infection prosthetic graft.

## INTRODUCTION

Vascular graft infection (VGI) is a feared complication that affects 2 to 4% of patients (1). It is associated with high morbidity and mortality rates of up to 75% (2, 3), and as many as 4 in 10 patients require amputation (4). Major complications include anastomotic disruption with massive bleeding, aortoenteric fistulas, distal embolization of infected thrombi, sepsis, and death (3).

Intraoperative contamination is considered the most common cause of VGI (3, 5), especially when the groin is involved (4, 6). Risk factors for infection may be host related, such as diabetes mellitus, immunosuppression, and obesity (7), or surgery-related, such as emergent procedures and reinterventions. Active infections in lower extremity wounds at the time of surgery also increase the risk (3, 5).

Up to 58% of VGIs are caused by Gram-positive bacteria, including Staphylococcus aureus, coagulase-negative staphylococci, and *Enterococcus* spp (8). Gram-negative bacteria are the next most common causative agents (38% of cases), followed by yeasts and anaerobic microorganisms (5).

VGI is a complex condition that requires multidisciplinary management. The main treatments are antimicrobial therapy and aggressive surgery involving complete graft removal and *in situ* or extra-anatomic reconstruction (9). Complete graft removal, however, is not always feasible due to the high risk of morbidity and mortality (3, 5). Antimicrobial therapy, often long term, is the cornerstone treatment (5), but its effectiveness can be limited by biofilm formation on the surfaces of grafts that cannot be removed (10).

Intraoperative measures to prevent graft infection are crucial and well established, but consideration must also be given to the type of graft used. Autologous material is preferable for vascular surgery (5), but it is often unavailable or unsuitable. Synthetic grafts are thus frequently used in peripheral artery interventions and aortic surgeries (5). They are designed to be as similar as possible to autologous graft material in terms of infection risk, patency, and user-friendliness.

Conclusive evidence is lacking on which types of grafts are most susceptible to infection (5). The aim of this study was to quantify the *in vitro* formation of methicillin-susceptible Staphylococcus aureus (MSSA) biofilms on the surfaces of biosynthetic and biologic grafts used in routine vascular surgery.

## RESULTS

The mean MSSA2 and MSSA6 counts (log_10_ CFU/mL), as well as standard deviation (SD) values, are shown in Table 1. Significant differences in biofilm formation were observed between the grafts (*P* < 0.001).

**TABLE 1:** Bacterial counts of biofilm formation by MSSA2 and MSSA6 on the surfaces of nine vascular grafts^*a*^

Material	Data for strain:			
	MSSA2		MSSA6	
	Mean (log_10_ CFU/mL)	SD	Mean (log_10_ CFU/mL)	SD
Dacron	7.30	0.34	7.51	0.25
Dacron-heparin	7.38	0.10	7.38	0.15
Dacron-silver	6.30	1.82	7.27	0.15
Dacron-silver-triclosan	0	0	2.42	2.44
Dacron-gelatin	7.55	0.24	7.47	0.15
Fusion	7.21	0.31	7.46	0.11
PTFE	6.40	0.39	6.88	0.31
Omniflow II	3.89	2.10	3.62	2.21
Bovine pericardium	6.98	0.30	7.35	0.26

a Log, logarithm; MSSA, methicillin-susceptible Staphylococcus aureus; PTFE, polytetrafluoroethylene.

The Dacron-silver-triclosan and Omniflow II grafts showed the least biofilm formation for both strains and were each compared with the other grafts. MSSA2 did not form biofilm on the Dacron-silver-triclosan graft (0 CFU/mL), and the mean count on the surface of the Omniflow II graft was 3.89 CFU/mL (SD 2.10). The mean growth of MSSA2 on the other seven grafts was 7.01 CFU/mL (SD 0.82). The differences in biofilm formation by MSSA2 were significant in both cases: 0 CFU/mL (*P* < 0.001) on the Dacron-silver-triclosan graft and 3.89 CFU/mL (*P* < 0.001) on the Omniflow II graft versus 7.01 CFU/mL on the other grafts.

The mean bacterial counts for the biofilms formed by MSSA6 were 2.42 CFU/mL (SD 2.44) on the Dacron-silver-triclosan graft and 3.62 CFU/mL (SD 2.21) on the Omniflow II. The mean growth on the other seven grafts was 7.33 CFU/mL (SD 0.28). The differences in the mean counts for the other grafts were significant: 2.42 CFU/mL (*P* < 0.001) for the Dacron-silver-triclosan graft and 3.62 CFU/mL (*P* < 0.001) for the Omniflow II versus 7.33 CFU/mL on the other grafts.

**FIG 1:**
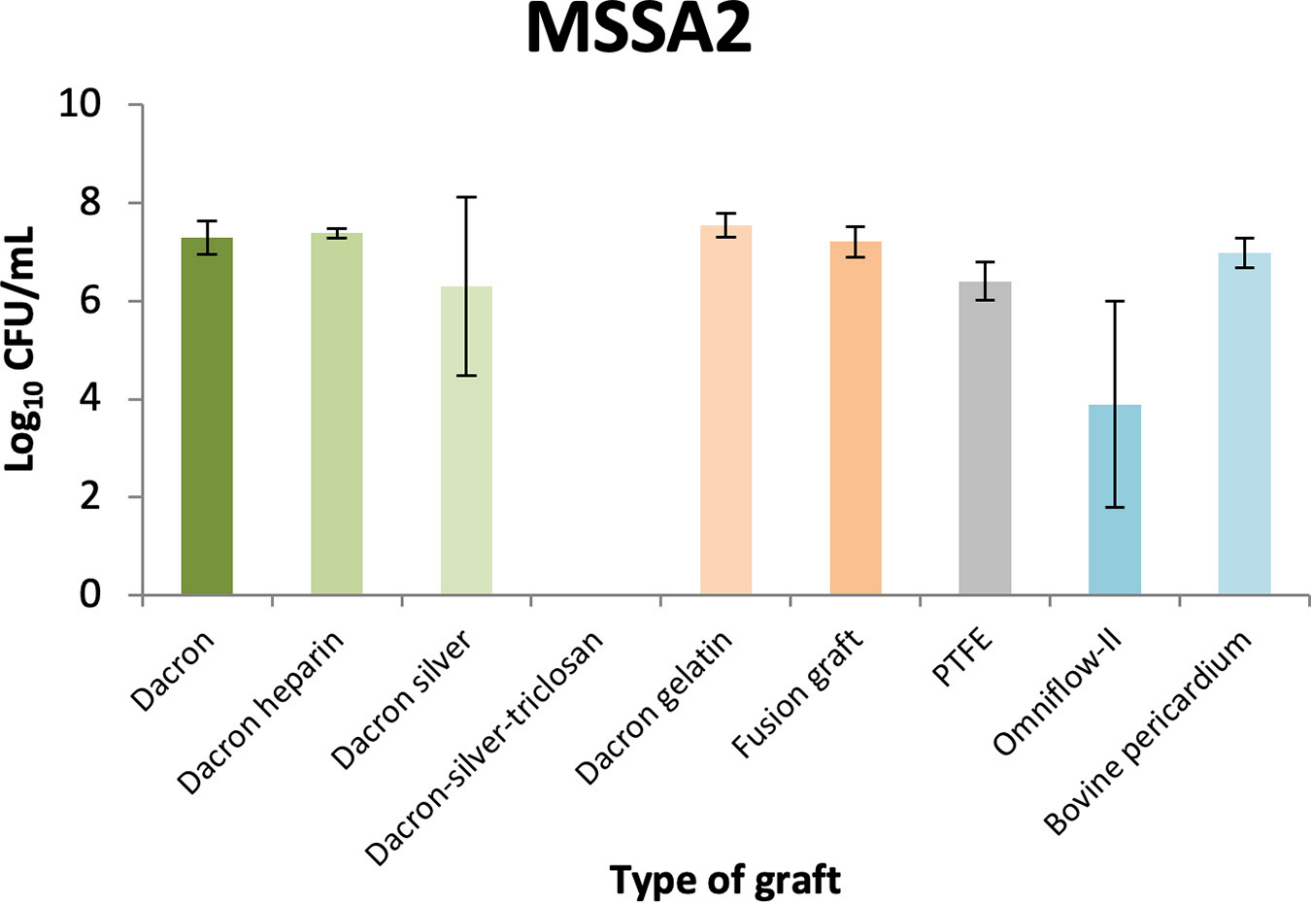
Biofilm quantification expressed as log_10_ CFU per milliliter and standard deviation (SD) of one strain of methicillin-susceptible Staphylococcus aureus (MSSA2) growing on the surfaces of nine different types of vascular grafts. The different grafts are indicated in different colors. The error bars in the graph indicate the standard deviation. log, logarithm; PTFE, polytetrafluoroethylene.

**FIG 2:**
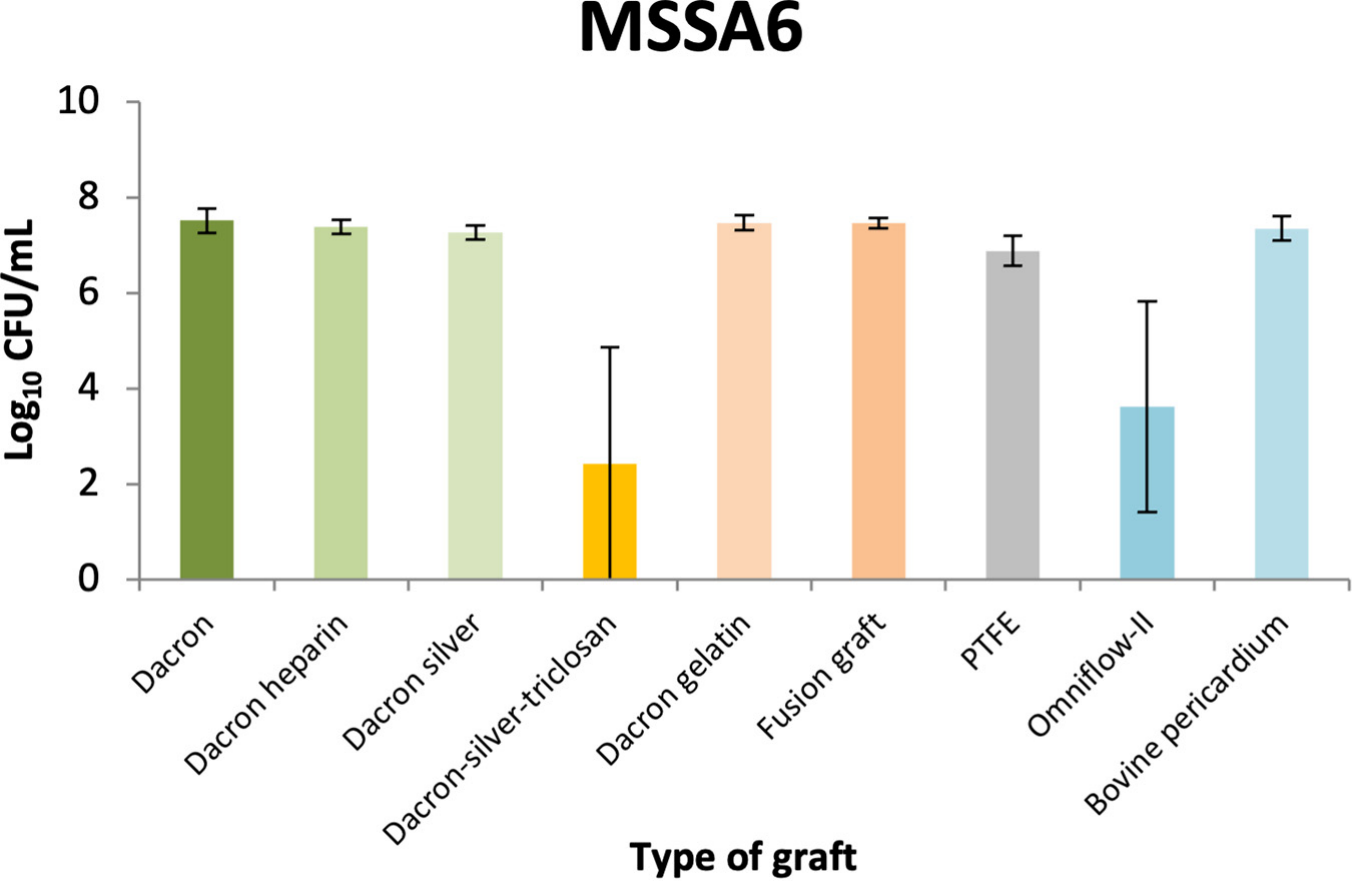
Biofilm quantification in log_10_ CFU per milliliter of one strain of methicillin-susceptible Staphylococcus aureus (MSSA6) growing on the surfaces of nine different types of vascular grafts. The different grafts are indicated in different colors. The error bars in the graph indicate the standard deviation (SD). log, logarithm; PTFE, polytetrafluoroethylene.

The combined results of all the experiments and the standard deviation (SD) values are shown in Fig. 1 and 2.

## DISCUSSION

In this *in vitro* study, we studied biofilm formation by two clinical strains of MSSA on the surface of nine vascular grafts used in routine clinical practice in Spain. Overall, the grafts that were most resistant to biofilm formation were the Dacron-silver-triclosan graft and the Omniflow II. The performance of the Dacron-silver-triclosan graft was particularly notable, as the bacterial count for one of the strains was zero in all experimental replicates.

The antimicrobial properties of silver acetate-coated grafts are enhanced by the addition of triclosan [5-chloro-2-(2,4-dichlorophenoxy)phenol]. The excellent results observed for the Dacron-silver-triclosan graft in our *in vitro* study confirm this improved efficacy and are consistent with recent reports (11–13). Ricco et al. (11) were the first authors to demonstrate the bactericidal activity of silver-triclosan against methicillin-resistant S. aureus (MRSA). Their findings were later corroborated by Berard et al. (12), who showed that grafts combining triclosan and silver acetate had better short-term antimicrobial activity against Escherichia coli, MRSA, and Candida albicans than those containing silver only. In a later study, they also showed that Dacron-silver-triclosan grafts had superior bactericidal efficacy to rifampicin-soaked grafts (13).

Omniflow II, a biosynthetic graft designed to minimize the risk of infection, has shown good patency rates (14). In our study, it was associated with significantly less biofilm formation than the other grafts analyzed, although other *in vitro* studies have reported contrasting results (15, 16). Our findings are more in line with promising reports of low reinfection rates in clinical settings (17, 18).

Staphylococcal infections are more common in peripheral vascular grafts, while Gram-negative infections are more common in intra-abdominal grafts; in the latter, an aortoenteric fistula should be suspected (19). Further work is needed to determine whether the results observed in this *in vitro* study would hold for different infection sites.

Grafts are also impregnated with antimicrobial agents to reduce infection risk (20). While rifampicin-impregnated grafts and silver acetate grafts have shown good results in experimental studies (21), considerable reinfection rates have been observed in clinical settings (5, 22).

In our study, no differences in MSSA biofilm formation were observed between the silver acetate-coated graft and the other grafts analyzed, confirming previous findings by Hernández-Richter et al. (23). We also observed no differences between the Dacron and polytetrafluoroethylene (PTFE) grafts. Other studies, however, have reported greater bacterial adherence to Dacron, possibly due to its porosity. Schmitt et al. (24), for example, described greater adherence to woven polyester (Dacron) than expanded PTFE for S. aureus, Staphylococcus epidermidis, and E. coli, and their findings were confirmed by *in vitro* assays by Herten et al. (2). Heparin did not modify biofilm formation, and the findings for the Intergard heparin graft were very similar to those observed for the Hemagard graft featuring Dacron only. We also observed no differences for the gelatin-sealed graft (Gelsoft), supporting previous findings by Yasim et al. (25). Similar to our results, Lumsden et al. (26) found no differences in biofilm growth between the Fusion graft combining Dacron and PTFE and the grafts featuring these components in isolation.

Bovine pericardium, which is widely used in cardiac surgery, is also used for peripheral artery surgery and, more recently, for *in situ* prosthetic reconstruction in patients with aortic graft infections (27–29). The use of bovine pericardium for reconstruction has been associated with low reinfection rates in a number of studies, although it should be noted that these studies had short follow-ups (27, 28, 30, 31). The MSSA strains in our study showed similar levels of biofilm formation on the bovine pericardium and synthetic grafts. Lorenz et al. (32), using bioluminescence imaging for the *in vivo* detection of S. aureus biofilms on vascular grafts in mice, showed that bovine pericardium was significantly less resistant to S. aureus infection than the other grafts studied.

A biofilm is defined as a community of microorganisms surrounded by a hydrated, extracellular matrix of polymeric substances (polysaccharides, proteins, lipids, and nucleic acids) formed by these microorganisms (2). The matrix allows the microbes to adhere to surfaces (biologic or synthetic), resulting in the immobilization of cells within the biofilm and providing mechanical stability and a barrier against the penetration of antimicrobials (33). In such cases, infections are difficult to resolve with medical treatment only.

Because biofilms confer increased protection against the immune system and antibiotics, the use of antimicrobial grafts could prevent these films from forming and limit colonization (11).

This study has some limitations. As an *in vitro* study, its findings cannot be simply extrapolated to clinical settings, since *in vivo* bacterial adherence is influenced by other factors, such as graft endothelialization, contact with plasma proteins, and cellular immune responses (34). We also analyzed just two strains of MSSA, although these are preliminary results from a broader line of research that includes new experiments with other species of Staphylococcus and Gram-negative bacteria.

### Conclusions.

The findings of this *in vitro* study suggest that Dacron-silver-triclosan and Omniflow II grafts have the greatest ability to prevent biofilm formation by MSSA. Further studies are needed to confirm our results and guide graft choice in patients at high risk of infection.

## MATERIALS AND METHODS

### Vascular grafts used for biofilm formation.

We studied all prosthetic vascular grafts available for use in clinical practice. There were nine grafts: seven synthetic and two biologic. These were comprised of (i) Dacron (Hemagard; Maquet, Baden-Württemberg, Germany), (ii) Dacron heparin (Intergard heparin; Maquet), (iii) Dacron with silver acetate (Intergard Silver; Maquet), (iv) Dacron with silver acetate and triclosan (Intergard Synergy; Maquet), (v) gelatin-sealed Dacron (Gelsoft Plus; Vascutek-Terumo, Inchinnan, Scotland, UK), (vi) expanded polytetrafluoroethylene (ePTFE) (Propaten with CBAS heparin surface; W.L. Gore Associates, Putzbrunn, Germany), (vii) ePTFE merged with Dacron (Fusion; Maquet), (viii) polyester with denatured sheep collagen (Omniflow II; LeMaitre Vascular, Inc., Burlington, MA, USA), and (ix) bovine pericardium (XenoSure; LeMaitre Vascular, Inc.).

The synthetic grafts were sterilized with gas at a low temperature, while the biologic grafts were prepared with 70% alcohol under sterile conditions.

**FIG 3:**
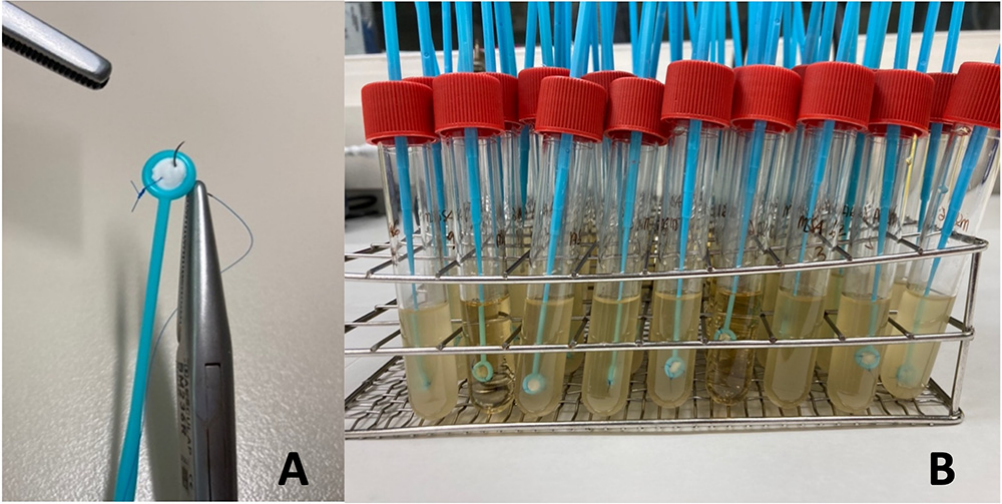
Graft preparation. (A) Graft piece attached to the loop by three stitches. (B) Samples were suspended in the tubes by perforating the caps.

The grafts were attached to a loop, as a modification of the protocol developed by Chandra et al., in order to submerge the samples in the inoculum and to keep them from floating. Using a standardized method, each graft was cut into circular pieces adjusted to the diameter of a 10-μL calibrated loop (Deltalab SL, Barcelona, Spain). Each graft piece was attached to a loop using 3 stitches of nonabsorbable monofilament polypropylene (Prolene 6-0; Ethicon Inc.; Johnson & Johnson, Somerville, NJ, USA) (Fig. 3A). The cap of a 10-mL tube was then pierced and the handle fitted so that the graft was suspended within the tube, 1.5 cm from the bottom (Fig. 3B).

Three replicates of each graft and MSSA strain were studied, and the experiment was performed in triplicate.

### Strains.

Two clinical strains of MSSA isolated from catheter infections were used for this *in vitro* study.

Quantification of biofilm formation was performed to ensure subsequent standardization. To this end, three MSSA strains (MSSA2, MSSA6, and MSSA7) were seeded on the surface of silicone and polyvinyl chloride discs using a slightly modified version of the protocol described by Chandra et al. (35). The biofilm formation steps involved a 90-min adherence phase, followed by a 24-h growth phase. Crystal violet staining according to the protocol described by Stepanovic et al. (36) was used to assess the biofilm-forming ability. MSSA2 and MSSA6 were the strongest biofilm producers and were therefore selected for this study.

### Biofilm formation on the surface of vascular grafts.

A slightly modified version of the protocol described by Chandra et al. (35) was used to characterize biofilm formation on the surface of the vascular grafts.

The MSSA strains were grown overnight in tryptic soy broth (TSB; Becton, Dickinson and Company, Le Pont-de-Claix, France) at 37°C and 60 rpm. After centrifuging and washing the bacterial suspension three times with sterile phosphate-buffered saline (PBS), pH 7.2 (Merck, Germany), an inoculum of 1 × 10^7^ CFU/mL was prepared with PBS, pH 7.2. Next, tubes containing 6 mL of the inoculum were prepared, and the vascular graft preparations were submerged in them. The tubes were incubated for 90 min at 37°C (adherence phase). Then, the vascular graft was gently and carefully moved from the adhesion step tube to the new tube containing 6 mL of TSB, avoiding adding any drops containing unattached microorganisms. The grafts were incubated for 24 h at 37°C and 60 rpm (growth phase).

Following the protocol of Chandra et al. (35), there were no washing steps between phases, in order to avoid underestimating the number of bacterial cells attached to the graft and therefore the biofilm formation.

### Quantification of biofilm formation.

The suture binding the graft to the loop handle was cut outside the tube on a sterile field, being careful not to transfer bacterial cells from the loop. Then, the graft was gently transferred to a sterile 12-well plate containing 1 mL of TSB using a sterile needle, avoiding adding any drops of liquid. Finally, both surfaces of the graft samples were gently scraped into the 12-well plate using the blunt edge of a no. 11 scalpel (Swann-Morton Ltd., Sheffield, UK). The suspension was transferred to new tubes, vortexed for 1 min, and sonicated for 10 min at 59 MHz. The tubes were centrifuged at 3,500 rpm for 10 min at room temperature and then resuspended with 1 mL of TSB; after that, the samples were serially diluted in physiological serum, and 50 μL was plated in tryptic soy agar (TSA; bioMérieux SA, Marcy l’Etoile, France) for quantitative culture. Finally, the bacterial cells were quantified and expressed as log_10_ CFU per milliliter.

### Statistical analysis.

Continuous variables are reported as the mean (standard deviation [SD]). Grafts were compared using analysis of variance, and differences were examined using *post hoc* multiple-comparison tests with Bonferroni correction.

Since none of the grafts could be considered controls, we decided to compare the two grafts with the weakest biofilm formation by both strains with the rest of the grafts. Differences were compared using the *t* test. A *P* value of <0.05 was considered statistically significant. The results are presented using descriptive statistics, and comparisons were made using SPSS version 26.0 (IBM Corporation).
